# Too Materialistic to Get Married and Have Children?

**DOI:** 10.1371/journal.pone.0126543

**Published:** 2015-05-08

**Authors:** Norman P. Li, Amy J. Y. Lim, Ming-Hong Tsai, Jiaqing O

**Affiliations:** 1 Singapore Management University, Singapore, Singapore; 2 Australian National University, Canberra, Australia; Tulane University School of Public Health, UNITED STATES

## Abstract

We developed new materials to induce a luxury mindset and activate materialistic values, and examined materialism’s relationship to attitudes toward marriage and having children in Singapore. Path analyses indicated that materialistic values led to more negative attitudes toward marriage, which led to more negative attitudes toward children, which in turn led to a decreased number of children desired. Results across two studies highlight, at the individual level, the tradeoff between materialistic values and attitudes toward marriage and procreation and suggest that a consideration of psychological variables such as materialistic values may allow for a better understanding of larger-scale socioeconomic issues including low fertility rates among developed countries. We discuss implications and describe how psychological factors relating to low fertility fit within evolutionary mismatch and life history theory frameworks.

## Introduction

*"May they combat the allure of a materialism that stifles authentic spiritual and cultural values*, *and the spirit of unbridled competition which generates selfishness and strife*.*"*

- *Pope Francis addressing youths in South Korea*, *August*, *2014*

In many modern societies, native populations are shrinking as citizens are delaying marriage and having less children. This is especially the case in East Asia, where countries like Singapore, Hong Kong, and South Korea have fertility rates that are far short of what is required to sustain a population. Although this trend seems to be related to economic development, relatively little is known—especially from a psychological perspective—regarding what it is about economic development that may be responsible for inducing aversions to marriage and procreation. In this paper, we considered the possibility that a key factor prevalent in modern societies—materialistic values—may negatively influence the value that individuals place on marriage and children.

### Economic Development

Differences in fertility rates between countries are significantly related to differences in economic development: people in more developed and industrialized countries tend to reproduce at much lower rates than those in less developed countries [1]. For instance, countries such as Niger and Mali are among the least economically developed countries, with per-capita GDPs of $800 and $1,100 [2], and estimated total fertility rates of 6.89 and 6.16, respectively [3]. Females in these countries, on average, are having many more children than what is required to sustain the local population. On the other hand, countries like Singapore and Hong Kong are among the most economically developed countries, with per-capita GDPs of $62,400 and $52,700 [2], and estimated total fertility rates of 0.80 and 1.17 [3], respectively. Females in these countries, on average, are falling far short of having enough children to sustain the local population.

Although quite telling, the association between fertility and economic development by itself is not a complete explanation for at least two reasons. First, it leaves unaddressed the significant differences in fertility rates among countries with similar levels of economic development. For instance, although Singapore and the United States are very similar in terms of gross domestic product per capita, Singapore’s total fertility rate (0.80) is the lowest in the world according to the CIA World Factbook, and less than half that of the U.S. (2.01) [3]. Second, although various demographic and socioeconomic explanations have been proposed (e.g., [4], [5]); it is still relatively unclear why, from a psychological perspective, people in modern societies (especially East Asian ones), who have all the benefits of modern healthcare and good living conditions, seem to be averse to getting married and having children—basic activities that are arguably at the top of the hierarchy of evolved human needs [6].

### Materialism and Attitudes toward Marriage and Children

Many explanations for low fertility and policy programs that have been offered thus far have focused on financial concerns over childbearing and childrearing, or the conflicts that women face between work and family [7], [8], [9], [10], [11], [12]. Examining these issues from a somewhat different angle, Li, Patel, Balliet, Tov, and Scollon [13] proposed that materialism—defined by *Merriam-Webster* as “a doctrine that the only or the highest values or objectives lie in material well-being and in the furtherance of material progress”—may be an important factor in explaining aversion toward marriage and procreation in the modern day and the observed differences between developed Asian and Western countries. That is, materialistic values, which are prevalent in developed countries and integral to the functioning of consumer-driven modern economies, may be so strong and require so much time and attention that they compete with and crowd out other values, including those relating to getting married and having children (also see [14]). This may be especially relevant in East Asia, where individuals report having greater materialistic values [15] and lower happiness and life satisfaction [16], [17], [18].

Looking cross-culturally, Li et al [13] found support for an Incompatibility of Materialism and Children model. Specifically, findings were consistent with the possibility that life dissatisfaction induces materialism, and both of these factors induce negative attitudes toward marriage and having children. Furthermore, Singaporeans were found to be more materialistic and less satisfied with life than Americans, thereby explaining the more negative attitudes toward marriage and children amongst Singaporeans versus Americans, and reflecting the tendency for East Asian countries to have lower fertility rates than equally developed Western countries.

#### The role of materialism

How might materialistic values—specifically, the pursuit of happiness and success based on the acquisition of markers of social status and other tangible goods—play such a central role in decreased fertility? The rapid globalization of consumer markets has brought about an unprecedented number and variety of products and services to modern countries. Although this is commonly considered a form of progress, the associated materialistic values, which have grown and flourished in this process, are nonetheless associated with a host of negative psychological factors, including negative affect [19], [20], psychological distress [21], worse mental health [22], and depression [23].

By competing with other values [24], materialism may also lead to a decreased desire for and ability to develop close, interpersonal relationships [25]. Indeed, people who encounter consumerism cues show decreased trust and increased competitiveness [19]. Individuals with materialistic values and attitudes place a lower emphasis on affiliative goals [22], [26], relational warmth [27], and close relationships [24]. Materialistic people also have greater social anxiety [28], insecurity, and tendencies to avoid intimacy [29].

Given the negative associations with forming interpersonal connections and establishing intimacy, materialism may be especially detrimental to people’s attitudes toward marriage and having children—endeavors that arguably require the most intimacy. In line with this reasoning, materialistic people are generally less satisfied with family life [30] and have more conflicts with romantic partners [31]. Couples where at least one partner is materialistic tend to face poorer marital outcomes than couples where neither partner is materialistic [32]. Spouses who are more materialistic are also more likely to perceive financial problems in their marriages [33] and regret having had children [34]. Furthermore, materialistic men are more likely to be childless than non-materialistic men [35]. Thus, materialism seems to be at odds with relational goals including getting married and having children.

In Asia, materialism seems to be stronger than in the West. Asia accounts for half of the 80 billion dollar market for luxury goods, exceeding the United States and Europe combined [36]. In Tokyo, 85% of women own a Louis Vuitton product [37]. Singaporean women have reported higher materialism-defined happiness (and lower life satisfaction) and have placed higher relative weight on a potential marriage partner’s social status than American women [13]. In a recent poll across 20 countries, a greater proportion of people from China than any other country endorsed the statements, “I measure my success by the things I own” and “I feel under a lot of pressure to be successful and make money” [15]. These values are present not only among adults but also among children. For instance, Chinese and Singaporean students seem to be more materialistic than American and Mexican students [38], [39]. Similarly, both Chinese and Japanese adolescents are more likely endorse the statement “Owning the right things is the most important thing in life” than American adolescents [40]. Luxury goods signal social status [41], [42], and social status may be a larger component of Asian versus Western culture and concepts of self-esteem [43]. Thus, social status concerns may drive consumerism and materialism more strongly in Asia than in Western countries [44].

### The Current Research

In the current research, we had two main objectives. First, we sought to test a model linking materialism to attitudes toward marriage and family. Li et al.’s [13] Incompatibility of Materialism and Children model established a link between materialism and attitudes toward marriage and family, but used only a single item to assess the importance that people place on having children. We sought to address this limitation by using multiple items to measure attitudes toward having children and by also directly measuring people’s intention to procreate. Our model also differs from the previous one in that we focused on materialism without the influence of life satisfaction as a factor. Thus, our Modified Incompatibility of Materialism and Children model proposes that materialistic values lead directly to negative attitudes toward marriage, which then lead to more negative attitudes toward having children, which in turn lead to wanting a lower number of children. We focused on exploring individual differences within Singapore, a modern country that exemplifies the prototypical East Asian profile of high per-capita GDP and low fertility.

Second, we sought to develop new materials for priming materialistic values and to test whether the kind of materialistic values that influence attitudes toward marriage and procreation can be readily induced. As others have also noted, experimental manipulations of materialism have been relatively rare. In an interesting investigation of women’s reactions to thin-ideal media, Ashikali and Dittmar [45] primed materialism by exposing participants to luxury advertisements. However, the researchers did not explicitly measure people’s materialistic values or attitudes, so it cannot be directly determined whether they were affected. Ku, Dittmar, & Banerjee [46] developed an effective materialism prime for children.

Bauer et al. [19] employed an interesting variety of situational cues for inducing consumeristic-materialistic mindsets. For one of the priming methods, they directly measured the effect of the prime on materialism. Specifically, they found that viewing a series of luxury advertisements versus natural scenes resulted in reporting greater materialistic goals. However, whereas those researchers measured extrinsic goals, we were interested in seeing if materialistic *values*—which arguably exist at a deeper level, can be externally influenced. Our own earlier efforts produced no differences in materialistic values between those who viewed luxury advertisements and those who viewed non-luxury control advertisements. Accordingly, we turned to recent research from others that has primed participants using personally engaging, vividly descriptive scenarios (e.g., [47]). Given that their methods have successfully induced fundamental human motives ranging from mating to self-protection, we sought to adopt them to activate materialistic values.

On the one hand, values are considered to be at the core of personal identity [48] and value systems may be relatively stable [49]. On the other hand, value systems may be subject to change depending on the situation [50] and some research indicates that values can be activated [51]. To see whether materialistic values can be externally influenced, we devised a new procedure—using established descriptive-scenario methods instead of mere exposure to advertisements—to prime luxury consumption at a deeper level, and examined its effects on materialistic values.

## Study 1

We first tested our Modified Incompatibility of Materialism and Children model relating materialism, attitudes toward marriage, attitudes toward children, and desire for children in both men and women. Because we were specifically interested in examining attitudes toward marriage and attitudes toward children as mediators of the association between materialistic values and number of children desired, we examined only the hypothesized indirect effects of materialistic values on number of children desired.

### Method

#### Participants

Participants were 91 undergraduates earning participation credit for psychology courses at a major Singapore university. There were 60 women (age: *M* = 20.52, *SD* = 1.14) and 31 men (age: *M* = 22.68, *SD* = 1.70). For both Studies 1 and 2, ethics approval was obtained from the Singapore Management University Institutional Review Board (SMU-IRB; approval #IRB-09-0097-A0099). Because the studies were conducted online, online consent was obtained instead of written consent. Specifically, participants could only proceed with each study and provide data if they indicated consent. This procedure for obtaining consent was approved by the ethics board.

#### Procedure and materials

Using Qualtics survey software, we presented participants with a series of surveys in random order.

#### Attitudes toward marriage

To measure attitudes toward marriage, we applied a uniform scale to a revised version [52] of the Favorableness of Attitudes Toward Marriage Scale [53]. Specifically, we used a 7-point Likert-type scale with one set of anchors (1 = very unlikely, 7 = very likely) for one question (“Do you think you will find, or have found, a person who is a suitable marriage partner for you?”), and another set of anchors (1 = not at all, 7 = very much so) for the other eight items (“How happy do you think you will be if you marry?”. Appropriate items were reverse-scored and a total score was computed for each participant (*α* = .84).

#### Attitudes toward having children

We measured desire for children with two items (*α* = .97): “Having children of my own (at some point) is important to me” and “I look forward to having children” (1 = strongly disagree, 7 = strongly agree).

#### Number of children desired

To obtain a specific measure of children desired, we asked participants the face-valid question, “How many children do you want?”

#### Materialism

We measured materialistic values with the Richins and Dawson [27] scale. In keeping with previous research [13], we combined a) the *possession-defined success* subscale (e.g., "Some of the most important achievements in life include acquiring material possessions") and b) the *acquisition as the pursuit of happiness* subscale ("I'd be happier if I could afford to buy more things") to capture materialism-based happiness and concepts of success (*α* = .83).

### Results and Discussion

**Fig 1:**
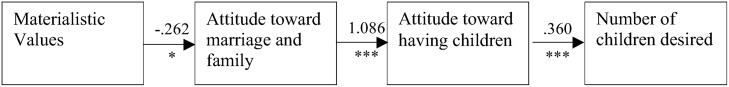
The Modified Incompatibility of Materialism and Children Model. *χ* ^2^ (3) = 2.74, *p* = .43, *CFI* = 1.00, RMSEA = 0.00, -0.006 < CI < -0.215 (indirect effect from Materialistic values to Number of children desired). Note: * *p* < .05, *** *p* < .001.

Neither participants’ materialism (*M* = 3.13, *SD* = 0.62), attitudes toward marriage and family (*M* = 3.47, *SD* = 0.73), attitudes toward children (*M* = 5.32, *SD* = 1.71), nor desired number of children (*M* = 2.24, *SD* = 1.05) differed by participant sex (*p*s > .17). Thus, we first collapsed the data across sex and tested the Modified Incompatibility of Materialism and Children model via path analysis using SPSS Amos. We had hypothesized that for young adults, materialism would negatively influence attitudes toward marriage, which would then influence attitudes toward having children, which would then influence the number of children desired. The model, shown in Fig 1 with unstandardized regression coefficients for each path, is a good fit to the data, *χ* ^2^ (3) = 2.74, *p* = .43. The results of the indirect effects demonstrated that materialism was negatively associated with the number of children desired via reduced favorable attitudes toward marriage that was adversely associated with positive attitudes toward having children (-0.006 < *95% Bootstrapping Confidence Interval* < -0.215, [54]). The results supported our hypothesis.

We reran the path analysis controlling for age and relationship status (not dating, dating). The model remained a good fit to the data, *χ* ^2^(3) = 3.27, *p* = .35, CFI = 1.00; RMSEA = 0.03. All predicted paths remained significant, as did the indirect effect from materialistic values to number of children, -0.25< 95% CI<-0.02. Still controlling for age and relationship status, we added participant sex as a moderator to the model and constrained the coefficients of the predicted relationships to be the same for men and women. The fit was reduced to a poor fit, *χ* ^2^(9) = 17.660, *p* = .039; CFI = 0.88; RMSEA = 0.10, suggesting that sex has significant moderating effects on the predicted relationships in the model. To better understand this moderating effect, we ran the model with the two control variables separately for men and women. The model was a good fit for women, *χ* ^2^(6) = 4.812, *p* = .568; CFI = 1.00; RMSEA = 0.00, but not for men, *χ* ^2^(6) = 8.421, *p* = .209; CFI = 0.88; RMSEA = 0.12.

## Study 2

In Study 2, we designed a luxury priming procedure. We then primed luxury (between-subjects: luxury, new control, old control) and measured materialistic values, attitudes toward marriage, attitudes toward children, and the number of children desired by men and women in Singapore. Once again, our path analysis focused only on the indirect effects of the hypothesized causal path from the first variable (materialistic values prime) to number of children desired.

### Method

#### Participants

Participants were 83 undergraduates earning participation credit for psychology classes at a major Singapore university. There were 54 women (age: *M* = 19.89, *SD* = 1.30) and 29 men (age: *M* = 22.00, *SD* = 1.54).

#### Procedure and materials

The same materials were used as in Study 1 but we also exposed participants to a priming condition (luxury, control) before presenting them with the surveys for materialistic values (*α* = .85), attitudes toward marriage and family (*α* = .83), attitudes toward having children (*α* = .97), and number of children desired. Following established methods for priming fundamental motives such as mating and self-protection (e.g., [47]), we developed a luxury reading passage vividly describing a person shopping for various luxury items on a large central street known for its shopping and high-end fashion. Because the sexes tend to prefer somewhat different luxury goods, we developed passages centering on different products for men (e.g., watches, cars) versus women (e.g., handbags, jewelry) to increase the relevance and thus, effectiveness of the passages. In the control condition, participants read about either an individual who is busy looking for lost keys—a procedure that also produces emotional arousal in participants [47], or a person taking a stroll and encountering various sights in a local park. Participants were asked to put themselves “in the shoes of the main character and experience the emotions that they are feeling”.

Participants then received a “booster shot” to help ensure that they remained in a primed state of mind, and to increase the effectiveness of the prime for those who may have only skimmed the reading passage. Adapting Meyer & Schvaneveldt’s [55] lexical decision paradigm, we presented participants in the experimental condition with luxury-related words that were present (e.g., “indulge”, “BMW”, “Chanel”) or nonwords in the respective passages that they read, and asked them to decide, as quickly as possible, whether the word being presented to them was a word that appeared in the passage. Participants in the control conditions performed the same task with descriptive words that were relevant to their respective passages.

### Results and Discussion

#### The effects of luxury priming on materialism

Using SPSS UNINOVA (GLM), we examined whether our priming materials had an impact on materialistic values. Materialism scores differed across the luxury prime conditions, *F*(2, 77) = 6.305, *p* = .003, *η* ^2^ = .14. Participants receiving the luxury shopping prime scored higher (*M* = 3.27, *SD* = 0.68) than those receiving the newly designed control condition passage (*M* = 2.76, *SD* = 0.53), *t*(81) = 2.76, *p* = .007, and higher than those receiving the previously validated [48] control condition passage (*M* = 2.76, *SD* = 0.48), *t*(81) = 2.90, *p* = .005. The two control condition passages did not differ in their effect on materialism scores (*p* = .999). Thus, in the subsequent path analyses, control condition primes were collapsed across the two types. There was no effect of participant sex (*p* = .881); nor did priming condition interact with participant sex (*p* = .746).

#### Other measures

Neither attitudes toward marriage and family (*M* = 3.37, *SD* = 0.74), attitudes toward children (*M* = 5.49, *SD* = 1.60), nor desired number of children (*M* = 2.36, *SD* = 1.11) differed by participant sex (*p*s > .23).

#### Materialism-marriage model

**Fig 2:**
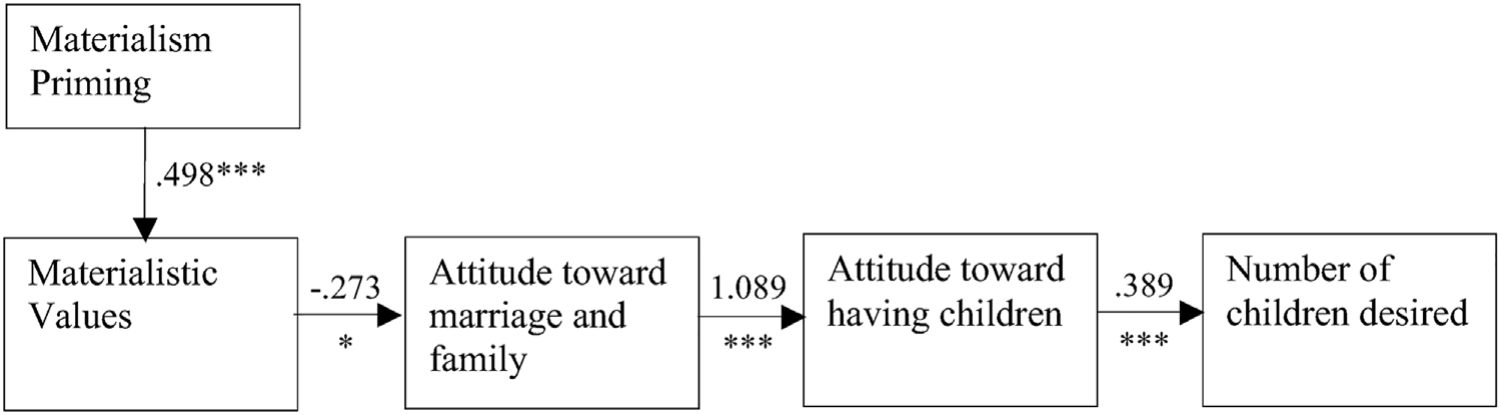
The Modified Incompatibility of Materialism and Children Model. *χ* ^2^(6) = 5.00, *p* = .54, *CFI* = 1.00, RMSEA = 0.00, -0.178 < CI < -0.001 (for indirect effect from Materialism Priming to Number of children desired). Note: * *p* < .05, *** *p* < .001.

Next, we collapsed participants’ responses across sex and tested the Modified Incompatibility of Materialism and Children model via path analysis. We had hypothesized that for young adults, the luxury priming manipulation would increase materialism, which would negatively influence attitudes toward marriage, which would then influence attitudes toward having children, which would in turn influence the number of children desired. The model, shown in Fig 2 with unstandardized regression coefficients for each path, is a good fit to the data, *χ* ^2^(6) = 5.00, *p* = .54. The results of the indirect effects demonstrated that luxury priming decreased the number of children desired by increasing materialistic values that negatively affect attitudes toward marriage that was adversely associated with positive attitudes toward having children (-0.178 < 95% Bootstrapping Confidence Interval <-0.01). The results replicated and extended the findings of Study 1 and supported our hypothesis.

We reran the path analysis controlling for age and relationship status (not dating, dating). The model remained a good fit to the data, *χ* ^2^(6) = 5.60, *p* = .47, CFI = 1.00; RMSEA = 0.00. All predicted paths remained significant, as did the indirect effect from materialistic values to number of children, -0.223 < 95% CI < -0.007. Still controlling for age and relationship status, we added participant sex as a moderator to the model and constrained the coefficients of the predicted relationships to be the same for men and women. The fit remained good, *χ* ^2^(16) = 17.18, *p* = .37; CFI = 0.99; RMSEA = 0.03, suggesting that participant sex has non-significant moderating effects on the predicted relationships in the model.

## General Discussion

Results of two studies supported our predictions. In our Modified Incompatibility of Materialism and Children path model, we suggested that materialistic values—the extent to which people derive happiness and status from material goods—would influence their attitudes toward marriage, which would then influence their attitudes toward having children, and then the number of children actually desired. The model fit the data well in both studies. Higher materialism was associated with more negative attitudes toward marriage and family as well as children, and a desire for less children. There was some evidence in Study 1, but not Study 2, that the model fit better for women than for men, though we caution against placing much weight on this sex difference, as it may be due to the low statistical power from having a relatively low number of male subjects but relatively high number of variables. Additionally, we developed a new luxury primary procedure that increased people’s materialistic values. This procedure may be useful in future research where materialism is being studied.

Taken together with previous research [13], our studies suggest that materialism is a key psychological process underlying why individuals in modern society might be increasingly delaying marriage and wanting less children. Perhaps by fostering a competitive rather than cooperative orientation and by utilizing key resources of time and energy, materialism may orient individuals away from forming close relationships with potential marriage partners and having children, which may require substantial amounts of not only financial, but also emotional, investment.

More generally, the research here further encourages a multidisciplinary approach to studying socioeconomic and policy-related issues. Although such issues are typically examined by economists and sociologists, we believe that evolutionary and social psychological perspectives may also be insightful. A consideration of research on the psychology of mating and marriage as well as consumer behavior may increase our understanding of the increasingly complex social world and how to approach its problems. Indeed, the research presented here is compatible with much of the work on low fertility that has been conducted by demographers and sociologists. Below, we describe two psychological paradigms rooted in evolutionary biology that may be particularly relevant to studying this area.

### Life History Theory

One applicable framework is life history theory [56], [57], which deals with key reproductively-relevant tradeoffs that organisms, including humans, make over their lifetime. A slow life history strategy is characterized by a “quality” approach to reproduction—organisms tend to invest in greater somatic development, take their time reproducing, have less offspring, and invest heavily in offspring. In contrast, organisms enacting a fast life history strategy employ a “quantity approach”—faster sexual maturation, more offspring, faster birth intervals, and lower parental investment.

Although originally used by evolutionary biologists to explain interspecies differences in reproductive strategy, life history theory has more recently been utilized in examining and explaining individual differences within species. In particular, harsh, dangerous, and unpredictable environments encountered early on in an organism’s development tend to promote fast life history strategies in that organism, whereas stable and resource-adequate environments with high social competition tend to promote slow life history strategies (e.g., [58], [59]) In this framework, individuals from countries like Singapore represent the slow end of a very slow species [60], [61], [62]. Furthermore, high population density and social competition in such countries induce a greater need to achieve and display social status, and may be among the key triggers that induce materialism and a slow reproductive rate.

### Evolutionary Mismatch

In conjunction with life history theory, evolutionary mismatch is another applicable framework. According to this perspective (e.g., [63]), human psychological mechanisms evolved over a period of one or two million years to adaptively respond to routinely encountered challenges to survival and reproduction. As such, the mechanisms are suited for the ancestral environment and may produce maladaptive results when they process much of the evolutionarily novel inputs of the modern world. For instance, tastes for sweet, salty, and fatty things likely evolved to impel our ancestors to eat fruits, nuts, and periodically encountered meat. In nature, these foods taste the best and provide the calories and nutrition necessary for survival. In the technologically advanced modern world, however, meat is readily available in mass quantities and foods are manufactured with unnaturally high amounts of sugar, salt, fat, and genetically modified chemicals. Thus, humans often prefer and overeat such foods over naturally occurring ones, leading to ailments such as tooth decay, diabetes, hypertension, heart disease, and hormonal imbalances [64], [65].

Similarly, modern-day materialism may involve a maladaptive engagement of mental processes evolved to impel individuals to acquire social status and signal their status to others. Although status signaling may be a manageable process in an ancestral village of 100 to 150 individuals, in the modern day, rapid technological advances combined with global competition induce symbols of social status to change at increasingly faster rates and luxury goods to lose their luster exceedingly quickly. Thus, it is not possible for most modern people to acquire what feels like enough status for very long or at all, and materialistic desires may lock individuals into a costly and futile pursuit of status targets perpetually moving upwards. Ironically, then, the pursuit of status may be leading to decreased reproduction in the modern day. Future research may benefit from incorporating theory and findings from this growing body of research.

### Limitations and Future Directions

The current research used traditional convenience samples and was conducted in one location. In future research, samples can be obtained in other Asian countries as well as Western countries to gauge the extent to which these results generalize or differ across countries. We also used path-analytic methods on survey data to investigate our directional predictions. To gain greater confidence in the each of the causal relationships, other methods can be utilized. Furthermore, although our luxury priming condition was balanced by two different control conditions, a more complete design would ideally include a “necessity” condition that takes individuals through the experience of shopping for non-conspicuous, non-luxury goods. Including such a condition would allow us to rule out the possibility that *any* shopping experience might increase materialism.

The current research helps open up questions for future work. For instance, given the links identified here and elsewhere [13], it is reasonable to ask whether interventions can be introduced to effectively curb materialistic values and thereby promote more positive values toward marriage and having children. A recent study suggests that this may be possible. Kasser et al. [66] implemented a financial education program and demonstrated that participation in the program reduced materialistic values and improved self-esteem over time. Future research can examine whether such programs are also useful in increasing people’s confidence in taking on marriage and family.

### Conclusion

As the quote at the beginning of the paper suggests, materialistic values may, from an early age, compete with and displace values relating to cooperation and interpersonal warmth. Such displaced values may directly or indirectly decrease the desire for marriage and family. Given the issue of declining local populations that East Asian countries are facing, and that materialism tends to be higher in these countries, researchers and policymakers may benefit from observing the psychological tradeoff between materialism and attitudes toward marriage and family that parallel macro-level tradeoffs between economic development and fertility.

## Supporting Information

S1 Data SPSS Data File Used in Analyses for Studies 1 and 2. (SAV)
